# Atlanta Academy of Medicine

**Published:** 1876-09

**Authors:** R. W. Westmoreland

**Affiliations:** Reporter


### Atlanta Academy of Medicine.

R. W. WESTMORELAND, M.D., REPORTER.

ATLANTA, July 4, 1876.

Meeting called to order by the President, Dr. H. V. M. Miller.

The following essay, by Dr. R. W. Westmoreland, was read:

### MEASURES ADOPTED FOR THE CURE OF ORGANIC URETHRAL STRICTURE.

As a means of exciting discussion upon a subject that every physician should be conversant with, I propose to advance some suggestions in this paper, and hope, in return, to hear opinions from each member of the Academy.

It is needless to say that a correct understanding of the nature of stricture is of primary importance. Without this one is acting in the dark, and more liable to do injury than good. This understanding we will suppose every physician has, and it is therefore deemed unnecessary to speak here in detail of the anatomy, pathology and complications of a disease upon which so much has been spoken and written. Being a disease which has excited so much interest and investigation here of late, descriptions sufficiently minute and frequent have afforded ample opportunity for a thorough knowledge of its bearings. I propose to discuss only the different modes of treatment, and shall give what I consider their advantages, and, where they have them, their disadvantages.

Dilatation, as being probably the first practiced and as possessing the mechanical requirement naturally indicated in the affection, shall first engage our attention. The importance of this procedure is suggested by the universal favor in which it is held by every one in every kind of treatment. Be the method incision, caustics, or rupture, the after treatment consists of the passage of instruments to maintain the calibre at the point of extension gained by the operation. Many rely with full confidence solely upon the gradual and almost imperceptible dilatation produced by an introduction of graduated bougies. This, it is said, effects a cure with less pain and more certainty than more severe remedies. The irritation excited by the presence of the instrument, judiciously applied, is of that degree most favorable to promote the absorption of the adventitious substance forming the stricture. This process going on gradually, eventually removes the obstruction entirely. This manner of proceeding, it is evident, will test the patience both of physician and patient, and cannot be practiced where time is precious.

Rupture, as another means, has its adherents, and reasons are adduced in support of this practice, equally, it seems, as efficient and plausible. The advantage upon which this mode is recommended is the readiness with which the obstruction is removed and troublesome symptoms relieved. Then, too, the tearing up of the structure is effected by inducing absorption, since a degree of excitement is produced commensurate to the purpose. Probably, too, the slight ulceration resulting from the laceration, and the consequent filling up of the rent by granulation, is the process to which the benefit is mainly attributable.

Oft recurring inflammations in the stricture produces, it is said, an elastic or resilient condition. In this type of the disease, since the dilatation treatment of itself is not capable of permanently reducing the contraction, internal incision has been recommended. This consists in dividing the stricture with some cutting instrument, made for the purpose, and then maintaining a desired calibre by the regular introduction of bougies.

This, for its merit, depends upon the same principle as that of rupture, since the wound made is left to granulate, and the contraction of the cicatrix controlled by the constant dilatation produced by the instruments. In this manner more surface is acquired, thereby enlarging the calibre, and much time saved in the treatment. The objection, however, to this plan is the liability to cut where it is not intended, and to do serious injury. This is more apt to occur where the operator is not well practiced with the instrument used in the cutting.

Again, we have the operation of external incision, practiced in strictures that are so tight that instruments of the smallest size cannot be introduced. In cases of this kind a probe is passed down to the obstruction, and then an incision is made down on the point of the instrument, and so on through the stricture. This, I would think, depended for its recommendation upon the question of the impermeability of the stricture. The truth of this idea has been denied by some of the best surgeons in the profession. They contend that no stricture is impassable with "*perseverance and sweet oil.*" This class of operating I would always steer clear of, when it is at all possible to use any of the others. The recovery is more prolonged, the chances against success more numerous, and the cure not at all more perfect than by simpler means.

Upon the idea that by inducing ulceration in the adventitious tissue, and thereby destroying the stricture, caustics were at one time used in the treatment. This, it would seem upon superficial consideration, a very good plan; but the difficulty of accurately applying the remedy, and even then of controlling the inflammation produced within proper bounds, has caused the remedy to come in to disuse.

These are the principal mechanical measures used in the treatment of urethral stricture. What now is to decide us in the choice of any one of them? In fact, is there any real intrinsic advantage that one has over the others? To the first inquiry I would say, circumstances and the fancy of the physician has more to do with the selection than anything else. To the second, the most reasonable answer would seem to be, that none has a universal and decided superiority. With gradual dilatation we can cure a stricture, probably, just as thoroughly, under favorable circumstances, as by any other mode. By rupture we at once arrive at the point which, by the more temporising treatment of gradual dilatation, it takes weeks to accomplish. By internal incision we please the patient more by sparing him the pain of the forcible dilatation, and yet have the immediate relief. For a treatment effective as based upon histological and mechanical principles, I myself am more inclined to favor rupture or incision.

The substance causing the obstruction is an exudation from the inflamed walls of the urethra. In agreement with Dr. Otis of New York, this inflammation is kept up by the urine so long as there is the least obstruction to its easy escape. Now it is given as a pathological fact that inflammation and absorption in mucous membranes are incompatable. This, it would seem, is the greatest objection to the gradual dilatation treatment, since the absorption of the substance is an item to success. By the rupture you may gain the condition necessary to produce the absorption, and at the same time remove the difficulty of obstructed urine, because the *inflammation* arising from the laceration soon subsides, ulceration sets up, and this result, as productive or associated with absorption, soon does away with the adventitious matter, or leaves a breach of sufficient dimensions to be, when filled by subsequent granulation, surface enough to form a normal calibre.

The instruments used for the purpose of forcible dilatation are various, and may all answer the purpose sufficiently well. Holt's, Voillermier's, Thebaud's, Thompson's, and Van Buren's may be mentioned as the principal ones, of which there are modifications and improvements. Sir Henry Thompson's divulsor I prefer, as combining the advantages of all the rest in one instrument. Passing this down over a filliform previously introduced through the stricture, the contraction may be enlarged to any extent desired, and is indicated by a scale at the handle of the instrument. When the constriction is ruptured, a steele bougie, the size of the normal urethra, may be introduced every two or three days, so as to make the granulations fill up, without contracting, the gaps made by the rupture. In this manner half the time is taken to perfect the cure that it takes in gradual dilatation. A few minutes only are required to give perfect relief from the more distressing symptoms, and the patient may be discharged to treat himself, without danger of false passages or other accident.

The discussion of the paper of Dr. R. W. Westmoreland being in order, Dr. John M. Johnson made a few complimentary remarks, and stated that as he was not a surgeon, was not prepared to discuss the merits of the paper.

Dr. W. F. Westmoreland, in reply to a question from Dr. Miller, as to his opinion as to the existence of impermeable stricture, and what he meant by impermeable stricture, remarked that he had more than once met with cases of impermeable stricture; that what he understood by impermeable stricture was cases in which we had a complete closure of the urethra from a destruction of the mucous membrane of this canal, from inflammation or other causes, and an adhesion of its walls, thus obliterating the canal at the point of stricture. Said he could not agree to any other definition; that it would not do to call a stricture impermeable because we had not succeeded in passing an instrument into the bladder. If such be the definition, what would be an impermeable stricture for one surgeon would not be for another. Or with the same surgeon, what would be permeable stricture one day, might be impermeable the next. Has invariably found impermeable stricture accompanied with urethral fistula. For its relief he has always practiced external urethrotomy, and as such strictures are usually found at the bulbous or membranous portion of the urethra, the operation has been perineal section.

Dr. Miller inquired how long he would wait in these cases of impermeable stricture and fistula before operating.

Dr. W. F. Westmoreland replied that he would be governed by circumstances. The indication is to perform the operation as soon as practicable, as the condition of the patient is constantly growing worse, and often his life is all the time more or less endangered. In all cases where the flow of urine is obstructed, and it has existed for any length of time, there is a tendency to catarrh of the bladder; the rule is, everything being equal, the greater the obstruction the greater this tendency to chronic cystitis. In cases, then, of impermeable stricture, with urethral fistulæ, and bladder complications, and particularly in cases where the obstruction to the flow of urine is sufficiently great to prevent the complete evacuation of the bladder at each effort, the cystic trouble will develop rapidly, frequently extending to the ureters and kidneys, producing a train of constitutional symptoms alarming in character. As soon as the subject can be prepared for the operation the better. Urethrotomy, under such circumstances, is far preferable to puncturing the bladder, as the latter will only give temporary relief, while the urethrotomy is permanent.

Dr. Bragg inquired of Dr. Westmoreland if he had ever seen a case af impermeable stricture without fistula.

Dr. W. F. Westmoreland replied that he had never seen a case of impermeable stricture without urethral fistula. His definition of impermeable stricture (complete occlusion by adhesions) makes fistulæ essential to preserve the life of the subject. He does not call a stricture impermeable when the urine passes through the canal even in drops. In a practical point of view, he said a stricture may be regarded as impermeable when, with the instruments at hand, it is found impossible to relieve the distended bladder by the introduction of an instrument. Such cases are sometimes seen unaccompanied with fistula, but they are not true impermeable strictures.

In reply to questions asked by Drs. Miller and Bragg, Dr. W. F. Westmoreland said that in cases of organic stricture of the urethra, where the bladder was, and had been, for a time greatly distended, the result of a partial or a complete obstruction to the flow of urine, and when with the instruments at hand it has been found impossible to relieve the distended bladder, and thus relieve the intense suffering and avoid the dangers of rupture, the operation is more imperatively demanded, and with less delay than in impermeable strictures. He never thinks of puncturing the bladder under such circumstances, but invariably performs perineal section.

Dr. Baird asked Dr. W. F. Westmoreland if he ever used the aspirator to relieve distended bladder, and under what circumstances.

Dr. W. F. Westmoreland said that he had frequently used the aspirator to empty the distended bladder from prostatic enlargements, and with satisfactory results.

Dr. Todd asked Dr. W. F. Westmoreland his opinion of spasmodic stricture—if he experienced difficulty in passing instruments through the urethra.

Dr. W. F. Westmoreland replied that he does not have the difficulty in that respect that he formerly did. Spasmodic stricture often depends upon the existence of organic stricture in some part of the canal. Mentioned a case in which the spasmodic condition was dependent upon irritation set up by a contracted meatus, and was relieved by the section of the meatus. The great difficulty he has had in the spasmodic stricture is in attempting to pass instruments too small. He selects as large an instrument as the condition of the canal will permit.

Dr. Bragg mentioned among the causes producing impermeable stricture, excessive venery, intemperate indulgence in alcoholic drinks, which, producing a kind of inflammatory condition, causes temporary impermeability. Thought that by perseverance an instrument of suitable size might be introduced. Inquired of Dr. W. F. Westmoreland if his experience did not lead him to think that impermeability in such cases was only temporary.

Dr. J. T. Johnson, in consideration of the fact of being engaged in the preparation of a paper in relation to the subject under discussion, curtailed his remarks so that he might present them more fully and to better advantage. Remarked that he believed stricture is occasionally met with in an impermeable condition. This is often caused by false passages, or inflammation set up, causing adhesion of the walls of the urethra. Favored external urethrotomy in preference to divulsion, as being less liable to produce shock. Gradual dilatation he prizes very highly as a means of treatment, and resorts to it under favorable circumstances.

Dr. Todd, in referring to the inquiry made by Dr. Bragg, asked Dr. W. F. Westmoreland if he thought spasmodic stricture was ever of temporary impermeability.

Dr. W. F. Westmoreland, not altogether understanding the point of Drs. Bragg and Todd, replied that he never failed to pass an instrument through the obstruction in spasmodic stricture. In enlarged prostate or inflamed organic stricture, he has failed even after persevering and repeated endeavors.

Dr. McIntosh, in reference to the impermeability from false passage, spoken of by Dr. J. T. Johnson, asked how this could cause impermeability; and if this were the case, would he operate immediately, and by what methcd.

Dr. Johnson replied that the false passage setting up an inflammation, would cause adhesions of the walls of the stricture. Would be governed by circumstances and urgency of the case in regard to the operation.

Dr. Miller inquired if it was necessary to cut when the instrument could be introduced into the bladder, even though it was by false passage.

Dr. J. T. Johnson thought that if an operation would open the natural channel, it were better than to have the unnatural one produced by the false passage. Divulsion he thought, when the condition of the patient permitted its use, should be practiced, more especially upon the membranous portion, and cutting upon the pendulous portion.

Dr. McIntosh inquired the reason of his preferring divulsion for the membranous, and cutting for the pendulous portion.

Dr. Johnson spoke of the greater ease with which the membranous portion could be divulsed, and the inconvenience of applying instruments internally for cutting.

Dr. McIntosh thought that the inconvenience of applying cutting instruments to the curve is done away with by using Maisonneure's instrument. Thought if divulsion were more dangerous than cutting, that it would be attended with less risk to use the cutting instrument in the curve, for it was in that portion of the urethra that operations, by whatever method done, were attended with most danger; while in the pendulous urethra they were comparatively harmless.

Dr. W. F. Westmoreland said that the character of stricture and condition of the patient should have more to do upon deciding the manner of treatment; it makes but very little difference as to the locality of the stricture. Has very often divulsed under favorable circumstances, and has never had a fatal case.

Replied to Dr. Todd as to indications against divulsion, and said where bad health and disease of the bladder exist, and where the patient has a very fast pulse, he would hardly divulse.

Academy adjourned.

